# Evidence of Human Parvovirus B19 Infection in the Post-Mortem Brain Tissue of the Elderly

**DOI:** 10.3390/v10110582

**Published:** 2018-10-25

**Authors:** Sandra Skuja, Anda Vilmane, Simons Svirskis, Valerija Groma, Modra Murovska

**Affiliations:** 1 Institute of Anatomy and Anthropology, Rīga Stradiņš University, Kronvalda blvd 9, Riga LV-1010, Latvia; Valerija.Groma@rsu.lv; 2 Institute of Microbiology and Virology, Rīga Stradiņš University, Riga LV-1067, Latvia; Anda.Vilmane@rsu.lv (A.V.); ssvirskis@latnet.lv (S.S.); Modra.Murovska@rsu.lv (M.M.)

**Keywords:** parvovirus B19, elderly, glia, PCR, immunohistochemistry, electron microscopy

## Abstract

After primary exposure, the human parvovirus B19 (B19V) genome may remain in the central nervous system (CNS), establishing a lifelong latency. The structural characteristics and functions of the infected cells are essential for the virus to complete its life cycle. Although B19V has been detected in the brain tissue by sequencing PCR products, little is known about its in vivo cell tropism and pathogenic potential in the CNS. To detect B19V and investigate the distribution of its target cells in the CNS, we studied brain autopsies of elderly subjects using molecular virology, and optical and electron microscopy methods. Our study detected B19V in brain tissue samples from both encephalopathy and control groups, suggesting virus persistence within the CNS throughout the host’s lifetime. It appears that within the CNS, the main target of B19V is oligodendrocytes. The greatest number of B19V-positive oligodendrocytes was found in the white matter of the frontal lobe. The number was significantly lower in the gray matter of the frontal lobe (*p* = 0.008) and the gray and white matter of the temporal lobes (*p* < 0.0001). The morphological changes observed in the encephalopathy group, propose a possible B19V involvement in the demyelination process.

## 1. Introduction

Due to increasing lifespan, the number of people suffering from neurological diseases in industrialized countries grows constantly. The aging of the human immune system is a continuous and active process involving mechanisms initiated by the response to infectious factors [1]. Infectious agents are often implicated in the development of neurological disorders, and human parvovirus B19 (B19V) has been shown to affect the immune system [2,3].

In addition to active infection causing tissue damage, many viruses have the ability to persist in the host cells in a latent state. This is typical of B19V, which usually remains quiescent in the host after primary exposure in early childhood and, thus, affects a high percentage of the human population. Lifelong tissue persistence of B19V genomic deoxyribonucleic acid (DNA) is a common outcome of B19V infection, confounding the interpretation of positive B19V DNA results in solid tissues [4,5,6]. After infecting the host, the viral genome persists in various organs, including the central nervous system (CNS) [7]. Lately, increasingly more studies report B19V-associated clinical syndromes with CNS manifestations, such as myalgic encephalomyelitis, progressive multifocal leukoencephalopathy, encephalitis, encephalopathy, or meningoencephalitis, both in immunocompetent and immunosuppressed patents [3,8,9,10,11,12].

Generalized demyelination reflects age-associated microstructural changes in the CNS of the asymptomatic elderly population, and provides insight into the neurobiology of aging [13]. In humans, viruses can cause demyelinating CNS diseases. In progressive multifocal leukoencephalopathy, demyelination seems to be a result of viral infection, dysfunction of oligodendrocytes, and failure of the immune system to control the infection [14].

The B19V genome encodes proteins, including the nonstructural protein NS1, and two structural proteins VP1 and VP2 [15]. Several intracellular pathways regulate the viral life cycle via macromolecular synthesis from the early pattern, characterized by prevalent expression of NS1 protein with its important regulatory functions, including control of the transcription and virus replication, as well as its contribution to host cell death, to the late pattern, characterized by prevalent expression of VP proteins [16,17]. The differentiation and physiological state of the cell, as well as the cell type, are critical for the virus to complete a productive cycle and induce apoptosis of the infected cell. Both structural VP proteins are produced and accumulated throughout the virus replication cycle [18,19].

B19V has a simple structure composed of the abovementioned proteins and a linear DNA molecule [20]. These non-enveloped viral particles are only 22 to 24 nm in diameter and show icosahedral symmetry, and often both empty and full capsids are detectable by negative staining electron microscopy [21]. Unfortunately, its size is very close to that of the ribosome in the cytosol and/or heterochromatin strands in the nucleus, which makes it difficult to distinguish between them, especially in the case of low viral load. Therefore, very few reliable images evidencing the presence of virus in human tissues have been obtained [22]. Detection of precise B19V localization in human tissues using electron microscopy is a challenge, but could provide valuable information about B19V and macroorganism interaction.

Although B19V has been detected in brain tissue by sequencing PCR products, little is known about its in vivo cell tropism and pathogenic potential in the CNS [23,24]. Information about the brain regions where B19V resides and its possible cellular targets is still lacking.

This study aimed to detect the presence of B19V and evaluate the spectrum and distribution of its target cells using brain tissue autopsy samples of elderly subjects. The following methods were used to achieve this goal: molecular virology and morphology, including optical and transmission electron microscopy.

## 2. Materials and Methods

### 2.1. Tissue Material and Sampling

Brain tissue autopsies of 48 individuals were included in the study with the aim to detect B19V DNA, antigens, as well as virions in brain cells. Brain tissue samples from the frontal and temporal cortex, and subcortical white matter selected from 24 elderly people with an unspecified encephalopathy (UEP) (mean age 63.9 (range 42–78); 12 males and 8 females) and 24 age-matched controls (mean age 61.4 (range 49–74); 16 males and 1 female) were used in accordance with the diagnostic criteria for UEP described in a previous study [25]. UEP-negative controls were chosen using the following inclusion criteria: (1) diagnosis of an encephalopathy was excluded by pathomorphological examination of the brain, applying measurements and calculations of the cerebral ventricle dimensions relative to the skull, as well as by microscopic evidence as described by Chapenko and colleagues [25]; (2) no prior history of neurological disorders, cerebrovascular disease, as well as traumatic brain injury, and no history of addiction-inducing substance use; and (3) common types of encephalopathy (metabolic, toxic, ischemic, and hemorrhagic) excluded during the lifetime and/or during autopsy. Upon tissue processing, the gray and white brain matter, obtained from temporal and frontal lobes, were sectioned. The determined post-mortem interval was between 7 and 30 h.

The study protocol and the use of the brain tissue autopsy samples were approved by the Ethics Committee of Rīga Stradiņš University (Decisions of the RSU Ethics Committee on No. 30.05.2013. and 2/30.03.2017.). Human tissue samples were taken in accordance with the tenets of the Declaration of Helsinki.

### 2.2. Real-Time Polymerase Chain Reaction

Total DNA was extracted from the tissue samples taken from the gray and white matter of frontal and temporal lobes. Standard phenol/chloroform extraction method was used. Concentration of extracted DNA was measured spectrophotometrically (Nanodrop ND-1000 Spectrophotometer, Thermo Fisher Scientific, Waltham, MA, USA) and the quality of DNA tested by detecting the presence of β-globin gene sequence using polymerase chain reaction (PCR) (C1000 Touch Thermal Cycler, Biorad, Hercules, CA, USA).

For quantitative detection of B19V genomic sequence, real-time PCR (qPCR) was performed in both the UEP and control groups. A commercially available qPCR kit (Parvovirus B19V Real-TM Quant, Sacace Biotechnologies Srl, Como, Italy), which included the standards, as well as the positive and negative controls, and the Biorad CFX96 Real-Time System (Biorad, Hercules, CA, USA) was used to detect the B19V VP1 genomic region, and the viral load was calculated as copies per microgram of DNA (copies/μg DNA). It is possible to detect a single copy/μg of DNA with this kit.

### 2.3. Immunohistochemistry

An immunohistochemical (IHC) reaction using anti-parvovirus B19 antibody (Millipore, Bilerica, MA, USA, clone R92F6, 1:150) that recognizes an epitope common to VP1 and VP2 structural proteins of B19V, and quantitative estimation of immunopositive cells was performed. Furthermore, after a routine examination, histological sections were selected and stained with the myelin basic protein (MBP, Santa Cruz Biotechnology, Inc., Santa Cruz, CA, USA, clone 1.B.645, 1:150), one of the major constituents of the myelin sheath to better visualize possible demyelination.

Formalin-fixed human brain tissue samples were processed conventionally using routine histopathology methods followed by immunohistochemistry and fluorescence microscopy. Paraffin-embedded histological sections of 4–5 µm were cut and mounted on HistoBond^®^+ (Marienfeld, Lauda-Königshofen, Germany) slides. Sections were deparaffinized in xylene and hydrated in a series of graded ethanol. The endogenous peroxidase activity was blocked with 30% hydrogen peroxide in methanol (30 min). For antigen retrieval, the sections were boiled in 0.01 M citrate buffer (15 min). Thereafter, consecutive sections were successively incubated overnight (4 °C) with the primary antibodies following the manufacturer’s recommendations. For visualization of antigen–antibody reactions, HiDef Detection™ HRP Polymer system (CellMarque, Rocklin, CA, USA) was used. After rinsing in phosphate-buffered saline (PBS) solution, sections were incubated with HiDef Detection™ Amplifier for 10 min at room temperature (RT) and HiDef Detection™ HRP Polymer Detector for 10 min (RT), respectively. The antigen sites were then visualized with 3,3′ diaminobenzidine (DAB) tetrahydrochloride kit (DAB+Chromogen and DAB+Substrate buffer, Cell Marque, Rocklin, CA, USA) applied for 5 min. The sections were counterstained with Mayer’s hematoxylin, washed with tap water, dehydrated, cleared, and mounted in Roti^®^ Histokitt (Carl Roth, Karlsruhe, Germany).

Cells that were labeled by the B19V antibody and displayed brown reaction products in nuclei and/or cytoplasm were considered immunopositive. Sections from cases with known antibody positivity were used as positive controls. Immunohistochemical controls that included substitution of the primary antibodies with tris(hydroxymethyl)aminomethane (TRIS) solution were used as negative controls.

The immunohistochemical analysis included evaluation of immunostained neurons, glial cells (astrocytes, oligodendrocytes, as well as microglia), and endothelial cells. The total number of immunopositive cells appearing within the microscopic field, reflecting a certain brain region, was estimated quantitatively. The expression of antigens was estimated using a Leica light microscope (LEICA, LEITZ DMRB, Wetzlar, Germany) and a Glissando Slide Scanner (Objective Imaging Ltd., Cambridge, UK) in 10 randomly selected visual fields of each sample (magnification 400×) representing the gray and white matter of the regions of interest. Reproducible measurements of tissue markers were obtained using the Aperio ImageScope program v12.2.2.5015.

For immunofluorescence, after immunostaining with primary antibody, sections were washed with PBS buffer (3 × 5 min), and then secondary goat anti-mouse IgG-FITC: sc-2010 antibody (Santa Cruz Biotechnology, Inc., Santa Cruz, CA, USA, 1:300) was applied. The sections were counterstained with 4′,6-diamidino-2-phenylindole (DAPI) (Thermo Fisher Scientific, Invitrogen, Loughborough UK, 1:3000) and embedded in Prolong Gold with DAPI (Thermo Fisher Scientific, Invitrogen, Loughborough, UK). Tissue autofluorescence was suppressed by 0.2% Sudan Black B solution (Sigma Aldrich, St. Louis, MO, USA) before coverslipping. For immunofluorescence sections, digital images were captured using a Nikon confocal microscope Eclipse Ti-E (Nikon, Brighton, MI, USA).

### 2.4. Transmission Electron Microscopy

Tissue autopsy material pieces (1 mm^3^) were processed for conventional transmission electron microscopy (TEM) and fixed in 2.5% glutaraldehyde in 0.1 M phosphate buffer 2–4 h (4 °C), postfixed in 1% osmium tetroxide (OsO4) 1 h (4 °C), and rinsed in PBS without sucrose. Samples were then dehydrated in a series of graded ethanols and acetone solutions, and embedded in epoxy resin (Sigma-Aldrich, Buchs, Switzerland). Ultrathin, 60 nm thick fine sections were cut with an LKB ultramicrotome, collected on Formvar-coated 200 mesh nickel grids, stained with 2% uranyl acetate and lead citrate, and examined in a JEM 1011 transmission electron microscope (JEOL, Akishima Tokyo, Japan) at magnifications of 8000× to 50,000×. Neuronal elements were identified in electron micrographs as described by Peters and colleagues (2004), and Bowley et al. (2010) [26,27].

For TEM examination, we used real-time PCR and immunohistochemically confirmed B19V-positive samples. For immunogold labeling, pretreatment using 0.01 M citrate buffer in a warming chamber (15 min, 98 °C) was applied [28,29]. To block free aldehyde groups, glycine in PBS for 30 min was used. In this step, and all following procedures, grids were placed with the tissue side facing downwards. To prevent nonspecific sticking of the immunoreagents, sections were incubated in blocking buffer (5% BSA, 5% normal goat serum, 0.1% cold water fish gelatin in PBS; pH 7.6) for 1 h (RT). Afterwards, incubation with primary antibody/incubation buffer (1% BSA, 1% normal goat serum, 0.1% TWEEN, 0.1% sodium azide in PBS; pH 8.2; 1:100) was used (overnight incubation at 4 °C). The immunogold labeling was performed by Protein A with small gold particles (10 nm) in the incubation buffer as the immunomarker (Ted Pella, Inc., Redding, CA, USA; 1:100). To remove unbound gold conjugate, sections were washed with PBS (3 × 15 min), then tri-distilled water, and dried for 30 min (RT). Finally, sections were stained with 2% uranyl acetate in water and lead citrate. Cellular structures were considered to be immunogold labeled when they were in direct contact with at least one colloidal gold particle and contained within a profile in which there were at least two gold particles, as recommended for the brain samples by Nirenberg et al. [30].

### 2.5. Data Analysis

Apart from descriptive morphology, statistical analysis was performed in order to estimate the immunohistochemistry results. To test whether the collected numerical data are normally distributed, the D’Agostino and Pearson, Anderson–Darling, and Shapiro–Wilk normality tests were applied. The comparison of means between different groups of numerical variables was performed using one-way ANOVA. Homogeneity of variances was tested using Brown–Forsythe and Bartlett’s tests and, in a case of unequal SDs, Brown-Forsythe and Welch ANOVA tests were applied. If data were not normally distributed, the comparison of medians between different groups was switched to non-parametric one-way ANOVA on ranks or Kruskal–Wallis test followed by two-stage step-up method of Benjamini, Krieger, and Yekutieli as post hoc test. The chi-square test was performed for categorical variables. To compare numerical values between two groups, the 2-tailed Mann-Whitney *U* test was applied, and relations between the numbers of immunopositive cells were investigated using Spearman’s correlations. The correlations were considered as follows: 0.2 to 0.4—weak, 0.4 to 0.7—moderate, and 0.7 to 0.9—strong. IHC results are expressed as violin plots, median and interquartile range (IQR) as dispersion characteristic, and *p*-value less than 0.05 (*p* < 0.05) was considered as statistically significant. In violin plots, the medians were used to represent the approximate ratio of visual fields (out of 240) with B19V-positive cells to fields with B19V-negative cells (“0”—ratio less than 1.0, “1”—more than 1.0). qPCR results were presented as geometric means with ±95% confidence interval (95% CI).

All the graphs (including violin plots), calculations, and statistical analyses were performed using the program GraphPad Prism 8 (demo, GraphPad Software, La Jolla, CA, USA).

## 3. Results

### 3.1. Real Time Polymerase Chain Reaction

**Figure 1:**
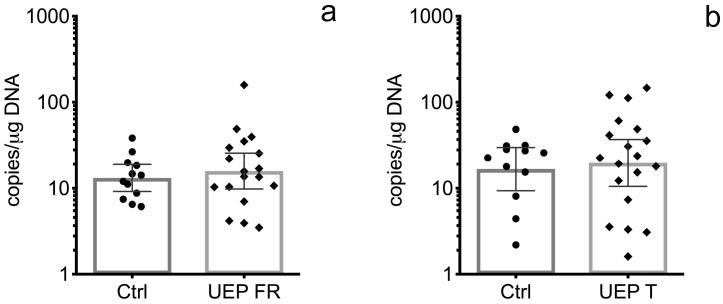
Representation of data from the brain tissue analysis: (**a**) Copies of B19V VP1 genomic sequence in the frontal lobes of control (Ctrl) and unspecified encephalopathy (UEP) groups; (**b**) Copies of B19V VP1 genomic sequence in the temporal lobes of control (Ctrl) and UEP groups. Scatter plots with bar represent geometric mean ± 95% CI.

In the UEP group, the B19V VP1 genomic sequence was detected in 18 out of 24 (75.0%) frontal lobe tissue samples, and in 19 out of 24 (79.17%) temporal lobe tissue samples, while in the control group, it was found in 15 out of 24 (62.5%) DNA samples extracted from the frontal lobe and in 14 out of 24 (58.33%) DNA samples extracted from the temporal lobe. The geometric mean value of the B19V load in DNA samples extracted from the frontal lobe was 15.91 (lower 95% CI—9.83 and upper 95% CI—25.76) copies/μg of DNA in the UEP group, and 14.60 (lower 95% CI—9.52 and upper 95% CI—22.37) copies/μg of DNA in the control group (Figure 1a). In DNA samples extracted from temporal lobe tissue samples, the geometric mean value of B19V load was 19.77 (lower 95% CI—10.54 and upper 95% CI—37.06) copies/μg of DNA in the UEP group and 16.80 (lower 95% CI—8.25 and upper 95% CI—34.22) copies/μg of DNA in the control group (Figure 1b). The difference between the B19V load in UEP and control groups was not statistically significant.

### 3.2. Immunohistochemistry

There was a small number of B19V-positive neurons in both groups—UEP as well as controls.

**Figure 2:**
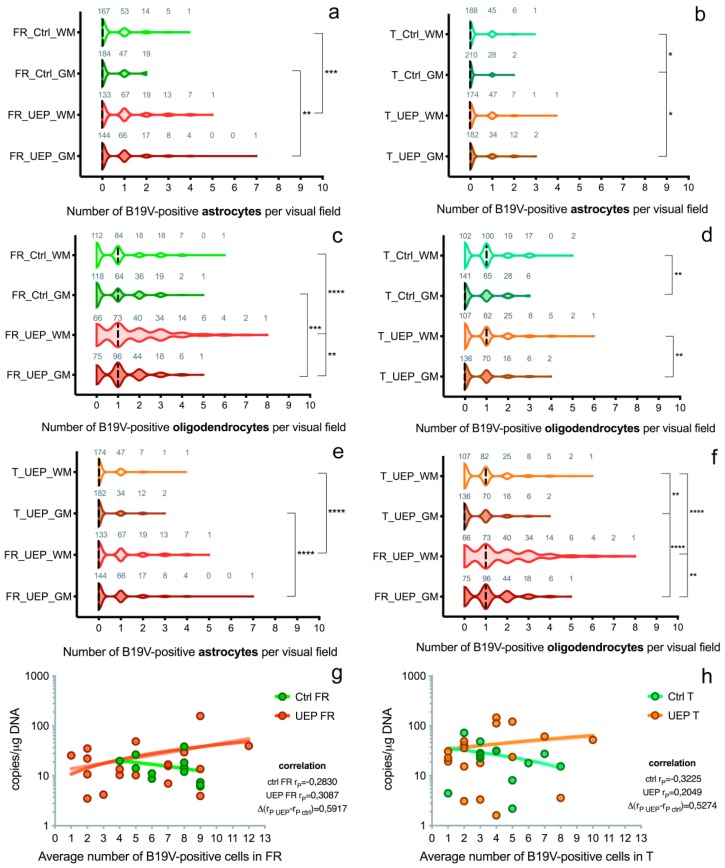
Data representing brain tissue analysis: (**a**) Distribution of B19V-positive astrocytes per visual fields in the gray (GM) and white matter (WM) of the UEP group and controls (Ctrl), frontal lobe (FR); (**b**) Distribution of B19V-positive astrocytes per visual fields in the gray (GM) and white matter (WM) of the UEP group and controls (Ctrl), temporal lobe (T); (**c**) Distribution of B19V-positive oligodendrocytes per visual fields in the gray (GM) and white matter (WM) of the UEP group and controls (Ctrl), frontal lobe (FR); (**d**) Distribution of B19V-positive oligodendrocytes per visual fields in the gray (GM) and white matter (WM) of the UEP group and controls (Ctrl), temporal lobe (T); (**e**) Distribution of B19V-positive astrocytes per visual fields in the gray (GM) and white matter (WM) of the UEP group, frontal lobe (FR), temporal lobe (T); (**f**) Distribution of B19V-positive oligodendrocytes per visual fields in the gray (GM) and white matter (WM) of the UEP group, frontal lobe (FR), temporal lobe (T); (**g**) Correlation of qPCR (*y*-axis) and IHC (*x*-axis) data in the frontal lobe (FR) tissue samples, comparison of regression slopes of the control and UEP groups; (**h**) Correlation of qPCR (*y*-axis) and IHC (*x*-axis) data in the temporal lobe (T) tissue samples, comparison of regression slopes of the control and UEP groups. Violin plots: dashed lines represent the approximate ratio of visual fields (out of 240) with B19V-positive cells to fields with B19V-negative cells (“0”—ratio less than 1.0, “1”—more than 1.0); numbers in gray show visual fields; asterisks represent a significance level (* *p* < 0.05, ** *p* < 0.01, *** *p* < 0.001, **** *p* < 0.0001) of differences between groups (Kruskal–Wallis test followed by two-stage step-up method of Benjamini, Krieger, and Yekutieli as post hoc procedure).

In the frontal lobe, a significantly greater number of B19V-positive astrocytes was found in the UEP group compared to the controls in both gray (*p* = 0.0034; the ratio between B19V-negative/-positive cell number of the UEP group/controls was 147/85 = 1.73) and white matter (*p* = 0.0008; the ratio between B19V-negative/-positive cell number of the UEP group/controls was 177/100 = 1.77). In the temporal lobe, a similar observation was made only in the gray matter (*p* = 0.014; UEP group/controls was 64/32 = 2) (Figure S1a,b). The ratio of visual fields with B19V-positive astrocytes to fields with B19V-negative astrocytes in the frontal lobe was 97/144 = 0.67 in UEP gray matter, 107/133 = 0.80 in UEP white matter, 66/184 = 0.38 in controls’ gray matter, and 73/167=0.44 in controls’ white matter (Figure 2a). The ratio of visual fields with B19V-positive astrocytes to fields with B19V-negative astrocytes in the temporal lobe was 48/182 = 0.26 in UEP gray matter, 56/174 = 0.32 in UEP white matter, 30/210 = 0.14 in controls’ gray matter, and 52/188 = 0.28 in controls’ white matter (Figure 2b). No statistical differences between the number of B19V-positive astrocytes in the white compared to the gray matter were found in either frontal and temporal lobes of UEP subjects. In controls, a significantly (*p* = 0.014) greater number of B19V-positive astrocytes was demonstrated in the temporal lobe when the white matter and gray matter was compared (60 and 32, respectively).

In the frontal lobe, significantly more B19V-positive oligodendrocytes were found in the UEP group compared to the controls in both gray (*p* = 0.001; the ratio between B19V-negative/-positive cell number of the UEP group/controls was 267/206 = 1.3) and white matter (*p* < 0.0001; the ratio between B19V-negative/-positive cell number of the UEP group/controls was 387/208 =1.9), but in the temporal lobe, no such observation was made. The ratio of visual fields with B19V-positive oligodendrocytes to fields with B19V-negative oligodendrocytes in the frontal lobe was 165/75 = 2.20 in UEP gray matter, 174/66 = 2.64 in UEP white matter, 122/118 = 1.03 in controls’ gray matter, and 128/112 = 1.14 in controls’ white matter (Figure 2c). In the temporal lobe, the ratio of visual fields with B19V-positive oligodendrocytes to fields with B19V-negative oligodendrocytes was 94/136 = 0.69 in UEP gray matter, 123/107 = 1.15 in UEP white matter, 99/141 = 0.70 in controls’ gray matter, and 138/102 = 1.35 in controls’ white matter (Figure 2d). In the frontal lobe (*p* = 0.008), as well as in the temporal lobe (*p* = 0.003) of the UEP group, a significantly greater number of B19V-positive oligodendrocytes was found in the white matter compared to gray matter. In the control group, significantly (*p* = 0.003) more B19V-positive oligodendrocytes were found only in the temporal lobe when the white matter and the gray matter were compared (199 and 139, respectively).

**Figure 3:**
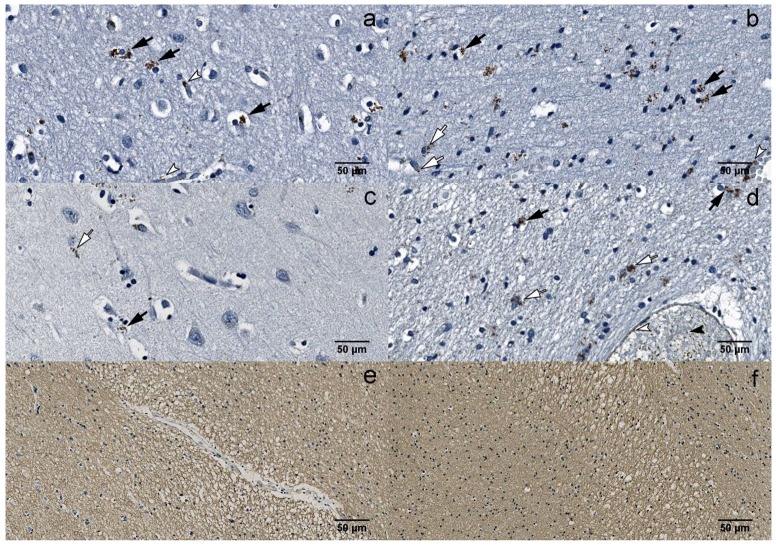
IHC: (**a**) B19V-positive oligodendrocytes (black arrows) and endotheliocytes (white arrowheads) in the gray matter of the UEP subject (frontal lobe, 400×); (**b**) B19V-positive oligodendrocytes (black arrows), astrocytes (white arrows) and endotheliocyte (white arrowhead) in the white matter of the UEP subject (frontal lobe, 400×); (**c**) B19V-positive oligodendrocyte (black arrow) and astrocyte (white arrow) in the gray matter of the UEP subject (temporal lobe, 400×); (**d**) B19V-positive oligodendrocytes (black arrows), astrocytes (white arrows), endotheliocytes (white arrowhead) and erythrocytes (black arrowhead) in the white matter of the UEP subject (temporal lobe, 400×); (**e**) myelin basic protein (MBP) immunoexpression in the white matter of the UEP subject (frontal lobe, 400×); (**f**) MBP immunoexpression in the white matter of the UEP subject (temporal lobe, 400×).

There were significantly (*p* < 0.0001) more B19V-positive astrocytes in the white and gray matter of the frontal lobe (177 and 147, respectively) compared to the temporal lobe (68 and 64, respectively) in the UEP group (Figure 2e and Figure 3a–d). Similar observations were made in the control group (*p* = 0.0061 and *p* < 0.0001, respectively). There were significantly (*p* < 0.0001) more B19V-positive oligodendrocytes in the white and the gray matter of the frontal lobe (387 and 267, respectively) when compared to the temporal lobe (192 and 128, respectively) in the UEP group (Figure 2f and Figure 3a–d). In the control group, similar observations were made only in the gray matter of the frontal lobe (206 and 139, *p* = 0.0061).

In the frontal and temporal lobes of UEP subjects, a significantly greater number (*p* ≤ 0.0001) of B19V-positive oligodendrocytes than astrocytes was found in the white matter. Similar observations were made in the gray matter (*p* ≤ 0.0001).

In the case of UEP, a statistically significant positive correlation was observed between B19V-positive oligodendrocytes in the gray and white matter of the frontal lobe (*r* = 0.487, *p* < 0.001). A similar correlation was found between B19V-positive astrocytes (*r* = 0.467, *p* < 0.001). Similar observations were made in the temporal lobe: the correlation between B19V-positive oligodendrocytes in gray and in white matter was *r* = 0.217, *p* < 0.001 and, for astrocytes, *r* = 0.315, *p* < 0.001, respectively.

There were significantly more (*p* < 0.001) activated microglial cells in the white matter when compared to the gray matter of both frontal and temporal lobes in the UEP group and controls. No significant differences between numbers of B19V-positive microglial cells in the white matter of the frontal and temporal lobes were found.

B19V-positive endotheliocytes were found in all slides studied, however, without statistically significant differences when numbers of cells were compared.

A weak positive correlation was established in cortices of the UEP group when qPCR data (amount of viral genomic copies) were compared to IHC (number of B19V-positive cells); the correlation was weakly negative in the control group (Figure 2g,h).

Multiple demyelinating lesions were detected in the white matter of the UEP group (Figure 3e,f).

**Figure 4:**
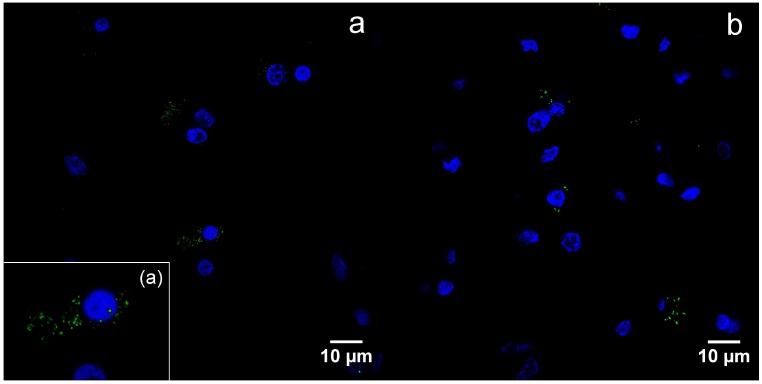

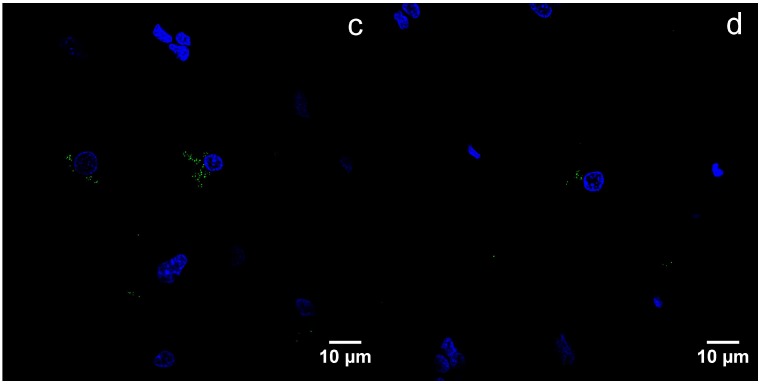
Detection of viral capsid antigens by immunofluorescence, confocal microscopy, DAPI—blue, B19V-immunopositive products—green: (**a**) B19V-positive oligodendrocytes and neurons in the gray matter of the UEP subject (frontal lobe, 1000×), the insert shows magnified view of the oligodendrocyte; (**b**) B19V-positive oligodendrocytes in the white matter of the UEP subject (frontal lobe, 1000×); (**c**) B19V-positive oligodendrocytes in the gray matter of the UEP subject (temporal lobe, 1000×); (**d**) B19V-positive oligodendrocyte in the white matter of the UEP subject (temporal lobe, 1000×).

By immunofluorescence, B19V capsid proteins were found in the cytoplasm, as well as nuclei of the oligodendrocytes (Figure 4a–d).

Overall, the immunohistochemistry results showed increased number of the B19V-positive oligodendrocytes in the white matter of the frontal lobe in the UEP group when compared to (1) the gray matter of the frontal lobe in the UEP group (*p* = 0.008), (2) the white and the gray matter of the temporal lobe in the UEP group (*p* < 0.0001), and (3) the white and the gray matter of the frontal and temporal lobes of the control subjects (*p* < 0.0001).

### 3.3. Transmission Electron Microscopy and Immunogold Staining

**Figure 5:**
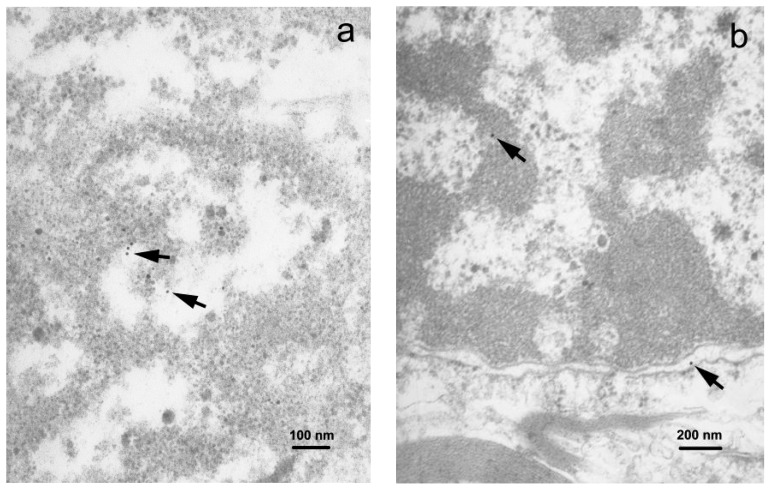
Immunogold staining, TEM: (**a**) Immunolabeled neuronal soma demonstrating the presence of the viral proteins in the nucleus (small dots showed by arrows, 50,000×); (**b**) (small dots showed by arrows, 30,000×). Nuclei show electron dense (dark) heterochromatin and electron lucid (light) euchromatin enclosed by nuclear envelope.

TEM examination of the gray matter demonstrated neuronal somata containing the rough endoplasmic reticulum (RER) and abundant free ribosomes (Figure S2a). Immunogold-labeled viral proteins were seen in the RER compartment and the nucleus, revealing predominance of fine chromatin (Figure 5a). Astrocytic soma showed irregular nuclear contours, and unevenly enlarged perinuclear space. The cytoplasm of astrocytes revealed moderate swelling and enlarged mitochondria. Gold particles were found within the nuclear compartment. The oligodendrocytes revealed round nuclei with clumped chromatin and few cytoplasmic organelles. Products of immunolabeling were found in the nuclei and cytoplasm of the oligodendrocytes (Figure S2b,c).

In the white matter, astrocytic somata and cell processes, identified by the presence of intermediate filaments, contained gold particles. Oligodendrocytes showed characteristic nuclei with clumped chromatin and an unevenly enlarged perinuclear space where viral proteins were found (Figure 5b). Thickened and split myelin sheaths, often with enlarged spaces between the myelin membranes, enveloped the axons. Some axons had intact myelin sheaths. The cytoplasm of these cells contained mitochondria that were slightly swollen and revealed the reduction of cristae. Myelin membrane-associated gold particles were found.

## 4. Discussion

In this study, we (1) detected B19V genomic sequences and measured viral load; (2) performed morphological analysis of brain tissue samples of UEP and control samples from elderly subjects; (3) analyzed the distribution of B19V antigen within different regions of the human brain; and (4) correlated the results with those of the qPCR.

The prevalence of B19V DNA in human brain tissue has been shown in several studies, including cohort studies examining dorsolateral prefrontal cortices and the cerebellum [23,31]. With a relatively high frequency, B19V DNA was found in the Hobbs et al. (approximately 42%) and Grant et al. studies (approximately 70%). These results are suggestive of B19V prevalence and demonstrated the presence of the B19V sequence (1) in 7/104 (6.7%) of the overall study sample, including unaffected controls—5/35 (14.3%) in the prefrontal lobe, and (2) in the cerebellum samples obtained from the Stanley Brain Collection, consisting of unaffected controls—68.6% of 35, schizophrenics—71.4% of 35, and subjects with bipolar disorder—67.6% of 34.

Our qPCR results show that B19V DNA is commonly found in brain tissue samples of individuals with UEP, as well as in the control group. This is consistent with the results of the abovementioned study. However, in both frontal and temporal lobe tissue samples, B19V genomic sequence was more frequently detected in the UEP group (75.0% in frontal lobe and 79.17% in temporal lobe) compared to the control group (62.5% in frontal lobe and 58.33% in temporal lobe), but no statistically significant difference was revealed. In addition to qPCR, we performed IHC to evaluate the location and spectrum of B19V-positive cells (Figure S3).

As described above, very limited data about the B19V target cells in the human brain have been reported. In this study, we have determined the localization of B19V in the human brain tissue cells using different morphological methods. We detected B19V in neurons, but numbers of B19V-positive cells were low. These results are similar to those demonstrated by Schaudien and colleagues [32]. They reported on occasional parvovirus DNA and antigen detection in the neurons, including in cerebellar Purkinje cells, and an association of parvovirus infection with leukoencephalopathy in dogs. Interestingly, we found varying dendritic spines in the B19V-infected cells: (1) presynaptic dendritic spines containing vacuolated mitochondria, cytoskeleton elements and fragments of RER, and (2) smaller and emptied postsynaptic dendritic spines similar to those reported by Carr and Sesack [33]. Structural reduction of synaptic density appears to be the normal process of aging, with the frontal cortex being especially affected [34].

Although it is a well-known fact that B19V penetrates human erythroid lineage cells [35], it has been demonstrated that B19V infects solitary endothelial cells in the fetal brain, endothelial cells of small myocardial blood vessels, and also a diverse range of non-erythroid lineage cells [6,36,37,38]. Our observations revealed the expression of the B19V protein in endothelial and activated microglial/macrophage cells of the frontal and temporal lobes. Clusters of microglial cells were a characteristic pathologic feature noted in the UEP group, in particular, in white matter, however, these were mostly B19V-negative. We found a greater number (*p* < 0.001) of activated microglial cells in the white, rather than the gray, matter of the frontal lobe. This leads us to believe that the white matter is more actively involved in the pathological process, in the case of UEP subjects. These results seem to be similar to the contention made by the Kerr group [39].

In the UEP group, a greater number of B19V-positive astrocytes and oligodendrocytes (*p* ≤ 0.0001) was found compared to the remaining B19V-positive cells. Furthermore, we found significantly (*p* ≤ 0.0001) more B19V-positive oligodendrocytes than astrocytes. These results are similar to those demonstrated by Schaudien and colleagues when animal parvoviruses were examined [32]. Moreover, we have found that the white matter was affected more strongly than the gray matter, particularly in the UEP group.

Viruses implicated in CNS diseases that include demyelination as a major feature of neuropathology, have been reported in numerous animal and human studies [14]. At the same time, it is known that the effects of ageing on myelin are complex, and include degenerative changes, as well as continuous myelin production throughout life [26,27]. We have shown a reduction of myelinated fibers in white matter, especially in UEP subjects (Figure 3e,f), similar to the SantaCruz et al. study on the John Cunningham polyomavirus in progressive multifocal leukoencephalopathy [40]. The results of this study indicated that, after primary injury and when virus antigen is abundant in oligodendrocytes, diffuse demyelination is observed [41,42]. We have confirmed the immunogold-labeling of the myelin sheath. Our data are in accordance with Dourmashkin et al. report on virus factories consisting of membranes that are involved in virus replication [22]. In our study, immunogold-labeling was a useful tool to improve detection of virus antigen, especially at low concentrations of the virus, as described by Goldsmith and Miller [43]. We hope that our observations have provided additional evidence confirming oligodendrocytes as the preferred B19V target.

Reviewing the literature, we found that B19V structural proteins enter the cell nucleus both early and later in the infection to enable virus replication and assembly [44]. Furthermore, observations by E. Mäntylä and collaegues have suggested nuclear envelope rupture and weakening of the nuclear lamina due to cleavage of the intermediate filament, in particular, lamin B2 by caspase 3 [45]. Our ultrastructural evidence appears to support this. However, capsids may also be exported to the cytoplasm or even out of the cell [46,47]. In this study, we found viral proteins in the nuclei of cells, as well as the cytoplasm. It has been observed that viral capsids in the cytoplasm directly alter the structure of the nuclear envelope, with particular damage to the outer nuclear membrane [48]. We observed a wavy nuclear membrane contour and an expanded perinuclear space in oligodendrocytes, along with gold labeling in the perinuclear space. A greater B19V-immnopositivity within oligodendrocytes and the ultrastructural changes described allow us to think that these are the cells where B19V resides and reactivation can occur.

In the current study, B19V was detected in both groups—UEP subjects and controls. However, we have detected a greater number of B19V-positive oligodendrocytes in the UEP group. These results are in accordance to those demonstrated by Hobbs when the presence of human parvovirus, in a large cohort, was studied [23]. At the same time, we agree with the statement that the spread of B19V was not related to immunosuppression, as we saw very few lymphocytes in the study group and the controls [49,50]. Our observations are similar to the histopathologic analysis of the biopsy specimen made by Nolan et al., which showed no evidence of perivascular lymphocytic infiltration in the frontoparietal lobe in case of human immunodeficiency virus infection [51]. Our B19V data in the UEP group, compared to controls, are consistent with the fact that the immune response varies in the elderly [1]. B19V appears to be a part of the normal aging process, as it was frequently detected in the control group. Despite the detection of B19V infection markers in the control group, but taking into account the higher frequency of B19V infection markers in the UEP group samples, we cannot exclude the possibility that B19V influences the more extensive demyelination observed in the UEP group. Further studies are necessary to prove or disprove this connection. In the future, more studies of B19V virus–target cell interactions must be developed, so that a better understanding of the molecular mechanisms of the interplay between the virus and the aging host may be gained.

The results were obtained by PCR, and confirmed ultrastructurally using immunogold-labeling of B19V, taking advantage of two distinct techniques. Analyzing the correlation of qPCR and IHC data, a rather large difference (Δ(rp UEP − rp ctrl) = 0.5274v − 0.5917) was found between the control and the UEP group; a change from negative correlation (control) to positive (UEP) was observed. Changes observed (a greater increase in viral load due to a higher number of B19V-positive cells) are much higher in the UEP group than in the control group, thus indicating a feasible role of B19V in UEP pathogenesis. Together with convincing IHC results, this could serve as a good argument to conclude that B19V has a definite role in UEP pathogenesis, despite the presence of this virus in the control samples. Finally, we fully agree with the statement that the frontal lobe, despite decades of intensive research by physiologists, anatomists, and clinicians, has remained the most mystifying of the major subdivisions of the brain [52].

## 5. Conclusions

This study aimed to investigate the involvement of B19V in the development of UEP in adult population using post-mortem brain tissue samples selected from elderly individuals with morphological signs of UEP.

Although B19V genomic sequence was more often found in the brain tissue samples of individuals with UEP than the brain tissue samples of individuals without signs of encephalopathy (controls), no statistically significant difference between the B19V load in brain tissue samples of frontal or temporal lobes was found when comparing UEP subjects to the control group.

In the white matter of the frontal lobe, an increased number of B19V-positive oligodendrocytes was found as compared (1) to the gray matter of the frontal lobe, and (2) to the white and gray matter of the temporal lobe. Our data demonstrate that the B19V invades the CNS with oligodendrocytes being the target cell, and this occurs with advanced age. The finding of immunogold-labeled viral proteins in the nuclei and cytoplasm of cells, in human brain tissues infected with the B19V, was a novel observation. By scanning the wider horizon now, the morphological approach gives the chance to acquire important additional data on the distribution of B19V, and correlation with pathological and clinical manifestations in the case of CNS disorders.

## Supplementary Materials

The following are available online at http://www.mdpi.com/1999-4915/10/11/582/s1, Figure S1: An overview of the B19V-positive cell numbers per visual field, Figure S2: TEM, Figure S3: Summarized representation of UEP-negative controls.
