# Supplementary material for: Evidence of Human Parvovirus B19 Infection in the Post-Mortem Brain Tissue of the Elderly
Source: Viruses. 2018 Oct 25;10(11):582. doi: 10.3390/v10110582 (PMC6267580; doi:10.3390/v10110582)
Supplement: Supplementary file 1 [file viruses-10-00582-s001.zip › Supplementary materials_S1_S2_S3/Figure S1.pdf]

Supplementary materials

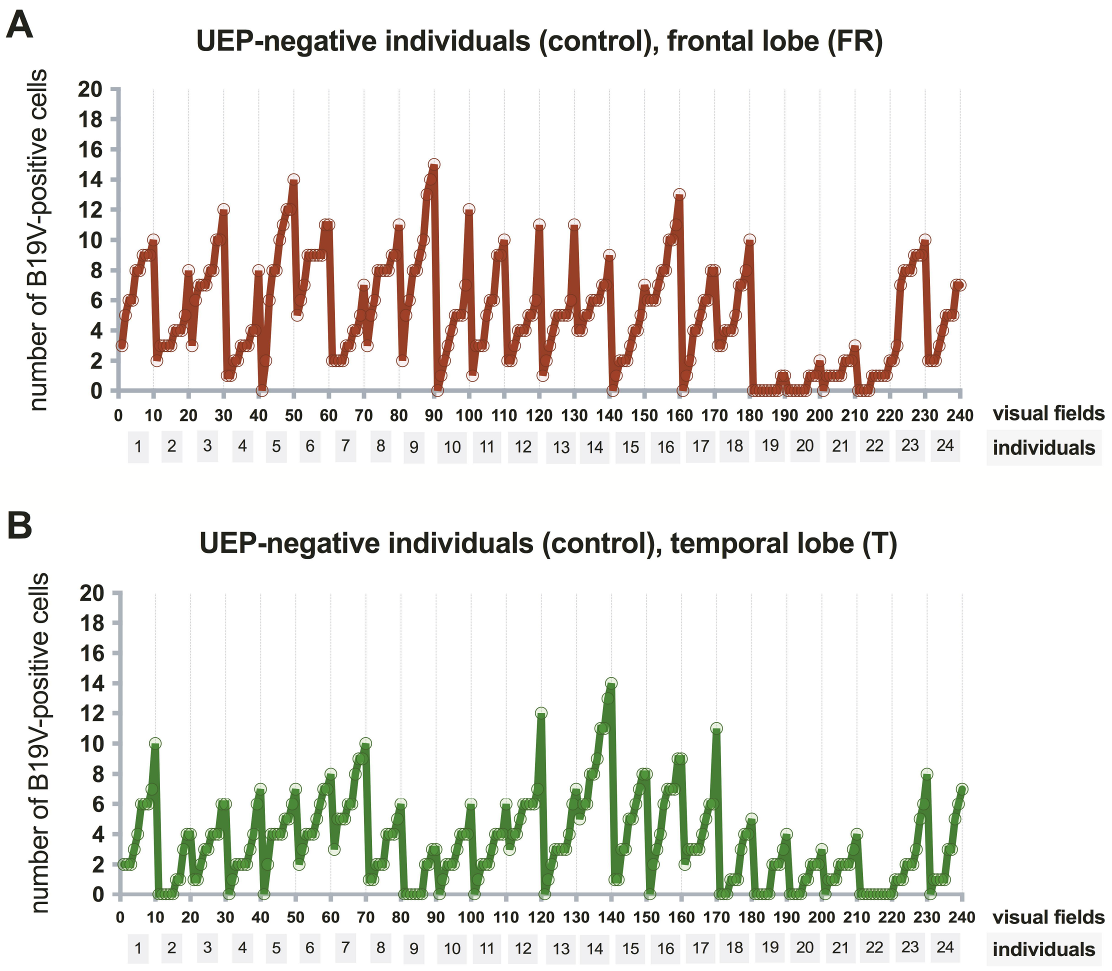

Supplementary Figure 1. An overview of the B19V-positive cell numbers per visual field: (A) Representation of the B19V-positive cell numbers per visual field in the UEP-negative control group in the frontal lobe, (B) Representation of the B19V-positive cell numbers per visual field in the UEP-negative control group in the temporal lobe.
