# Supplementary material for: Evidence of Human Parvovirus B19 Infection in the Post-Mortem Brain Tissue of the Elderly
Source: Viruses. 2018 Oct 25;10(11):582. doi: 10.3390/v10110582 (PMC6267580; doi:10.3390/v10110582)
Supplement: Supplementary file 1 [file viruses-10-00582-s001.zip › Supplementary materials_S1_S2_S3/Figure S2.pdf]

Supplementary materials

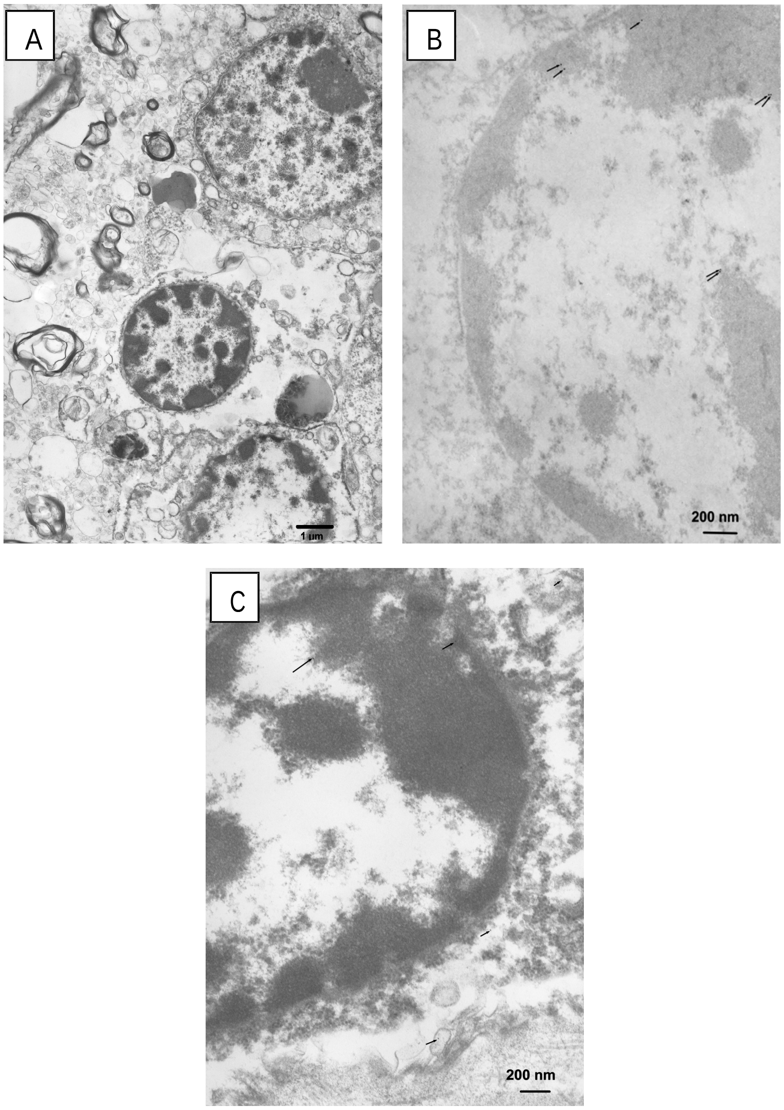

Supplementary Figure 2. TEM: (A) Electron microscope photomicrograph showing nuclei of the astrocyte, oligodendrocyte and neuron, original magnification ×50000, (B) Immunogold staining, oligodendrocyte, original magnification ×20000, (C) Immunogold staining, oligodendrocyte, original magnification ×20000. Figures (B) and (C): arrows show small gold particles (practically non-visible in this magnification) in the oligodendrocytes nuclei.
