# Supplementary material for: Evidence of Human Parvovirus B19 Infection in the Post-Mortem Brain Tissue of the Elderly
Source: Viruses. 2018 Oct 25;10(11):582. doi: 10.3390/v10110582 (PMC6267580; doi:10.3390/v10110582)
Supplement: Supplementary file 1 [file viruses-10-00582-s001.zip › Supplementary materials_S1_S2_S3/Figure S3.pdf]

Supplementary materials

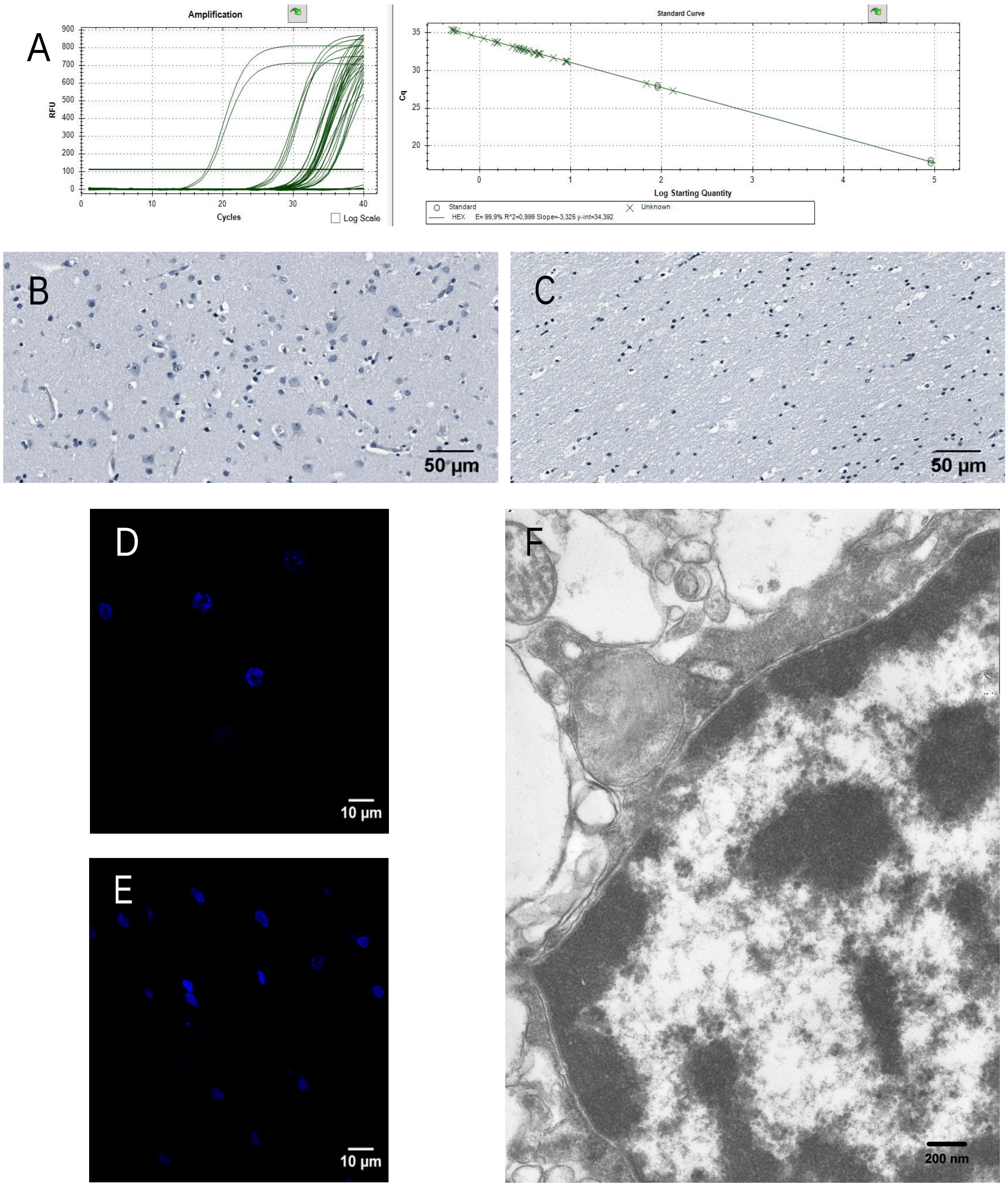

Supplementary Figure 3. Summarized representation of UEP-negative controls (controls, Ctrl): (A) Amplification and standard curves from B19V qPCR run. Standards, the positive control and B19V-positive DNA samples cross the threshold between the cycles 20 and 35, where the negative control and B19V-negative DNA samples do not reach the threshold level before the cycle 35; (B, C) Histopathology micrographs demonstrating the gray (B) and white (C) matter found in the frontal lobe of the subject without encephalopathy; both lack decoration of the viral capsid proteins stained brown by the precipitated DAB reaction product (original magnification ×200); (D, E) Immunofluorescence captures obtained from the frontal lobe of the subject without encephalopathy demonstrating nuclei of neurons and oligodendrocytes stained with DAPI (blue) and localized in the gray (D) and white (E) matter, respectively (original magnification ×1000); (F) Fine structure of the oligodendrocyte cytoplasm and nucleus revealed using transmission electron microscopy (original magnification ×20000).
